# Tocotrienols as an Anti-Breast Cancer Agent

**DOI:** 10.3390/antiox10091383

**Published:** 2021-08-29

**Authors:** Madison Trujillo, Anupreet Kharbanda, Christa Corley, Pilar Simmons, Antiño R. Allen

**Affiliations:** 1 Division of Radiation Health, University of Arkansas for Medical Sciences, Little Rock, AR 72205, USA; mtrujillo@uams.edu (M.T.); clcorley2@uams.edu (C.C.); pgsimmons@uams.edu (P.S.); 2 Department of Pharmaceutical Sciences, University of Arkansas for Medical Sciences, Little Rock, AR 72205, USA; akharbanda@uams.edu; 3 Neurobiology & Developmental Sciences, University of Arkansas for Medical Sciences, Little Rock, AR 72205, USA

**Keywords:** tocotrienols, breast cancer, γ-tocotrienol, δ-tocotrienol, natural adjuvant, anti-cancer

## Abstract

In the past few years, breast cancer has become the most prevalent type of cancer. The majority of patients receive combinatorial chemotherapy treatments, which may result in increased risk of developing drug resistance, a reduced quality of life, and substantial side effects. Treatment modalities that could lessen the physical toll of standard treatments or act in synergy with chemotherapeutic treatments would benefit women worldwide. Research into tocotrienols has thus far demonstrated their potential to be such an agent, with tocotrienols surpassing the pharmacological potential of tocopherols. Further research using in vitro and preclinical breast cancer models to support clinical trials is needed. This review uses bibliometric analysis to highlight this gap in research and summarizes the current and future landscape of tocotrienols as an anti-breast cancer agent.

## 1. Introduction

In 2020, female breast cancer surpassed lung cancer to become the most commonly diagnosed cancer worldwide [1]: 11.7% of 19.3 million new cases diagnosed in 2020 [1]. While being the fifth leading cause of cancer deaths globally, it is the foremost cause of cancer death in women [1]. Moreover, about 1 in 8 women is diagnosed with breast cancer, and in the U.S., the incidence of breast cancer has escalated each year since 2007 [1,2]. Hereditary and genetic factors account for 5–10% of breast cancer cases; nonhereditary risk factors linked to menstruation, reproduction, oral contraceptive use, hormone replacement therapy, alcohol consumption, and anthropometry more significantly influence incidence rates [3,4]. Standard treatments include surgery, radiation, chemotherapy, hormone therapy, and targeted therapies. Recent estimates project that 64.2% of women with node-positive breast cancer receive combination chemotherapy treatments [2]. Overall, the 2017 Global Burden of Disease Study showed that breast cancer incidence rates increased while mortality decreased between 1990 and 2017 [3]. While advances in diagnostic methods and treatments have led to a decrease in breast cancer mortality [5,6], patients continue to suffer from debilitating side effects and reduced quality of life. Short-term side effects of chemotherapy are nausea, vomiting, lethargy, hair loss, weight changes, and increased susceptibility to infection [7]. In addition, chemotherapy can induce infertility, organ damage, secondary cancers, and chemo brain that persist for years after treatment [7]. Long-lasting adverse effects of endocrine therapy include menopausal symptoms, osteoporosis, joint and muscle pain, increased risk of thrombosis, and endometrial cancer [8]. Not only does breast cancer and its accompanying treatments take a physical toll, they are also psychologically and financially challenging. Breast cancer survivors, compared to non-cancer patients, consistently report lower health-related quality of life up to two 2 years post-diagnosis [9]. Reduced health-related quality of life is also associated with an increased risk of mortality [10]. In 2017 alone, nearly 2 million cases of breast cancer were reported globally, corresponding with an estimated 17,708,600 years of “healthy life” lost [3]. To compound the issue further, breast cancer is the costliest type of malignancy to treat; in the United States in 2010, breast cancer treatments cost $16.5 billion [11]. The more effective but more expensive treatments developed since 2010 have only continued to drive treatment costs up [11].

The use of plant-derived medicine is not new: four well-known classes of anticancer agents are plant derived—camptothecin derivatives, epipodophyllotoxins, taxanes, and vinca alkaloids [12]. The use of natural products as adjuvants is only an extension of this practice. However, it has been acknowledged that pharmaceutical companies have prioritized new chemical techniques over exploring the use of natural products [13]. The ever-increasing incidence rate of cancer and the growing population of patients with a history of cancer show the inefficacy of this approach. Recent studies demonstrated that a variety of natural compounds act on the same molecular targets or physiological pathways as chemotherapeutic drugs do but with minimal adverse effects [14]. Natural compounds can increase the therapeutic efficacy of chemotherapeutic agents and reduce their toxicity [12,14]. The National Cancer Institute identified about 3000 plant species with potential anticancer activity [12]. While research into the anticancer properties of natural compounds has been rekindled, this integrative approach has not been incorporated into standard treatments [12]. One group of natural compounds in particular, tocotrienols, has demonstrated significant potential as an anti-breast cancer agent [15,16,17,18,19,20,21,22]. Known for their potent antioxidant activity, the innate anticancer properties of these unsaturated isoforms of vitamin E are often overlooked. This review will summarize current evidence and the potential of tocotrienols as an anticancer agent.

## 2. Materials and Methods

### 2.1. Bibliometric Data Source and Search Strategy

Web of Science (Clarivate Analytics, Philadelphia, PA, USA) was used to obtain the bibliometric data in June of 2021. This global citation database was used to retrieve scholarly articles and academic literature [23,24]. Four separate “advanced searches” (See Table 1) of the Web of Science Core Collection were run with the following parameters: Topic set (TS) = (insert topic terms), language: (English), and document types: (Article or Abstract of Published Item or Review). The key search terms were as follows:

(1) Search 1 = (tocotrienol OR tocotrienols) AND TS = (breast cancer);
(2) Search 2 = (tocotrienol OR tocotrienols) AND TS = (breast cancer AND chemotherapy);
(3) Search 3 = (tocopherol OR tocopherols) AND TS = (breast cancer);
(4) Search 4 = (tocopherol OR tocopherols) AND TS = (breast cancer AND chemotherapy).

With these settings, the software recognized co-occurring words in related articles. Book chapters, proceedings papers, and retracted publications were excluded from the search results. For each search, the full records and cited references were downloaded and saved in a tab-delimited file format. Downloaded Web of Science citation data included publication by institution, country, funding agency, and author.

### 2.2. Bibliometric Data Analysis and Presentation

VOSviewer version 1.6.16 (Centre for Science and Technology Studies, Leiden University, Leiden, The Netherlands) was used for network analyses. For each search, data was imported and analyzed separately. A thesaurus file was used to merge terms, account for spelling differences, or exclude vague terms (Table S1). This file was based on a list of the top 5000 words from the 450 million-word Corpus of Contemporary American English [25]. Additional terms such as “vitamin e” and “vitamin-e” or “trf” and “tocotrienol rich fraction” were added to the list, allowing them to be recognized as the same term. When generating the networks, the following options were selected: “create a map based on bibliographic data”, “read data from bibliographic database files”, “type of analysis: Co-occurrence”, “unit of analysis: all keywords”, and “counting method: full counting”. An individual term, whether appearing a single time or multiple times in a single publication was counted as one occurrence. Each term met or exceeded the minimum threshold of 5 occurrences. After analysis, terms were separately visualized as spheres with the terms from each search arranged in a network. Sphere size was proportional to the number of occurrences (the larger the sphere, the higher the number of occurrences). Curved lines between terms depict the number of co-occurrences (the closer the proximity, the higher the frequency). The colored scale illustrates the year of publication.

## 3. Background

### 3.1. Vitamin E

Initially known as “antisterility factor X”, the importance of vitamin E was first recognized in 1922 when Herbert Evans and Katherine Bishop noticed that leafy greens and small amounts of wheat germ oil corrected infertility in rats [26]. They pinpointed a new vitamin as the causative agent, with wheat germ oil being its highest source [26]. This vitamin, given the official name of vitamin E, is a mixture of 8 lipophilic isomers—4 saturated tocopherols and 4 unsaturated tocotrienols. All of these homologs have chromanol rings, which limit the flexibility of the molecules due to the bicyclic benzopyran structure [27]. The chromanol rings differentiate isomers by the number of methyl groups of each ring; the alpha (α) isoform has 3 methyl groups; the beta (β) has 2; the gamma (γ) has 2, and the delta (δ) has 1 [28]. The amphipathic molecules have differing hydrophobic side chains, with the tocopherols possessing a saturated side chain and tocotrienols having an unsaturated side chain. Plant-based oils are the primary natural sources of tocopherols and tocotrienols (collectively referred to as tocols), though nuts, seeds, grains, and vegetables contain varying amounts. Shahadi and Costa de Camargo summarized tocol content in underutilized edible oils [29].

### 3.2. Tocopherols

**Figure 1:**
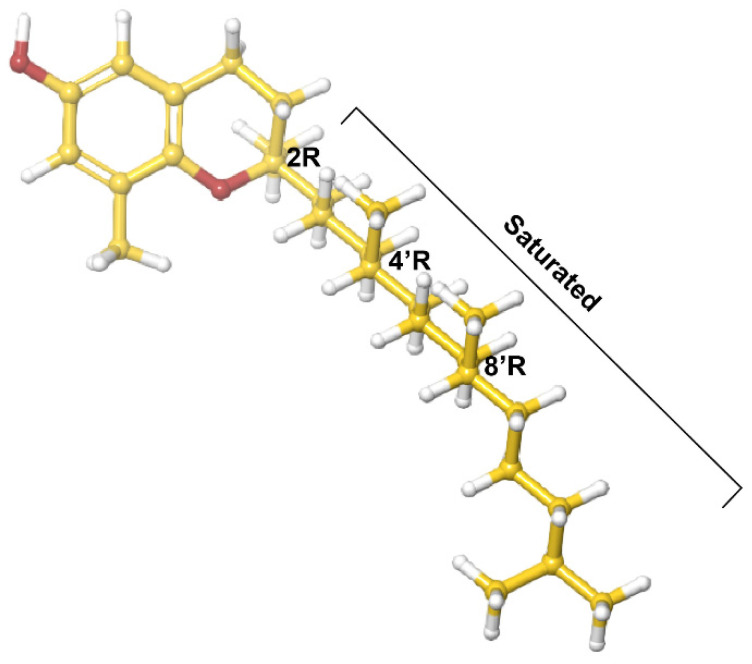
δ-Tocopherol.

The more studied tocopherol homologues have a longer, saturated phytyl tail compared to that of tocotrienols [30], the isomers of which have common 3-chiral stereocenters—C-2, C-4′ and C-8′ (Figure 1). Alpha-tocopherol is the most bioactive form, and along with γ-tocopherol, is the most common dietary form. It is worth noting that biological activity is unrelated to antioxidant activity [31]. One determinant of biological activity of an isomer is an affinity for α-tocopherol transfer protein (αTTP) [31]. This protein, primarily expressed in the liver, binds and improves the transfer of α-tocopherol between membranes and stimulates its release from hepatic cells [31]. Hosomi et al. demonstrated that αTTP preferentially binds α-tocopherol over the other tocopherol analogs and that there is a linear relationship between the relative affinity of tocopherol analogues and their biological activity [31]. At the same time, tocopherol supplementation has been shown to interfere with the bioavailability of other isoforms [32,33] Studies in the past two decades demonstrated that the isomers have distinct biological activities, and that the anticancer and anti-inflammatory activity of α-tocopherol is inferior to other, less-studied, isoforms [34,35].

### 3.3. Tocotrienols

**Figure 2:**
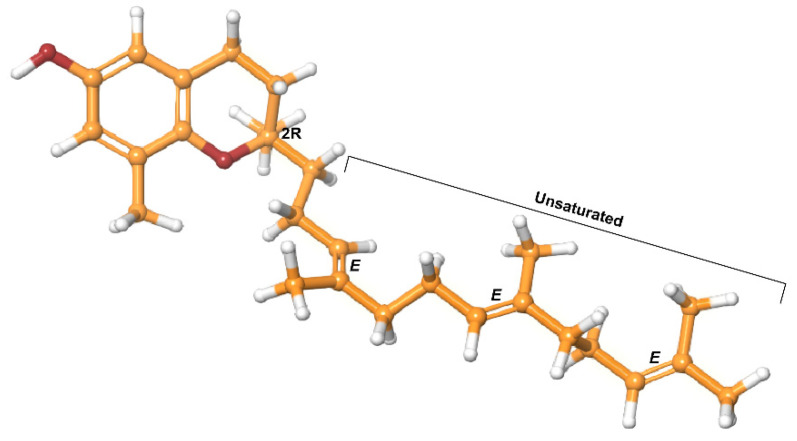
δ-Tocotrienol.

In contrast, tocotrienols have a shorter, unsaturated isoprenoid side chain [30]. They have a 1-chiral stereocenter (2R), with 3 double bonds taking the trans configuration (Figure 2). Studies continue to demonstrate that tocotrienols have at least the same potent antioxidant capacity as tocopherols [36,37]. However, tocotrienols are less readily available [17,38]. Tan, credited with discovering the 3 most abundant natural sources of tocotrienols, noted the tocopherols–tocotrienols ratio to be 50:50 in rice bran, 25:75 in palm oil; and 0.1:99.9 in annatto oil [38]. Of the tocotrienols, α-tocotrienol has the highest bioavailability, followed by γ-tocotrienol then δ-tocotrienol [39]. Even though the affinity of tocotrienols for αTTP is inferior, they cross cell membranes more efficiently due to their unsaturated isoprenoid side chain [20,37,39]. In addition, studies investigating absorption and bioavailability suggest that tocotrienols may use pathways independent of αTTP [33,40,41].

Tocotrienols have been shown to have therapeutic value in female reproductive health, cancer treatment, liver protection, skin protection, bone resorption, metabolic syndromes, cardiovascular health, and neurological disease [15,20,21,28,34,39,42]. Furthermore, tocotrienols possess functions independent of their antioxidant status that tocopherols do not have—particularly anticancer activity, chemosensitization, and neuroprotection [33,42,43]. In particular, the γ- and δ-tocotrienol isoforms have more profound anticancer properties (Figure 2) [18,22]. Between its inimitable functions and higher efficacy, tocotrienols have great promise as anticancer agents.

## 4. Model Relevancy

### 4.1. Current Tocotrienol Research

While extensive research from in vitro studies to clinical trials has been conducted with tocopherols, the same cannot be said for tocotrienols. To a lesser extent, the mechanism of action of tocotrienols has been investigated in various types of cancer cells [16,17,21,44,45] as well as in breast cancer cell lines specifically [18]. For example, Alawin et al. demonstrated that γ-tocotrienol accumulates in lipid rafts, suppressing human epidermal growth factor receptor 2 (HER2) signaling in SKBR3 and BT474 human breast cancer cell lines [46]. Takahasi and Loo assessed the apoptotic effects of γ-tocotrienol in MDA-MB-231 human breast cancer cells [47]. Pierpaoli et al. investigated the anticancer effects of alpha-, gamma-, and delta-tocotrienols in human and murine HER-2/neu breast cancer cells compared to alpha-tocopheryl succinate [48]. A few years later, their group demonstrated the antitumor activity of annatto tocotrienols in HER-2/neu transgenic mice [49]. A few dozen studies probed the effects of tocotrienols in breast cancer cells, but the vast majority of these, including the aforementioned studies, did not assess tocotrienols in the presence of chemotherapeutic agents in the treatment of breast cancer.

Additionally, some studies have suggested that tocotrienols have chemosensitizing potential. In breast cancer cells, the chemosensitizing actions of tocotrienols in combination with chemotherapeutic drugs including gefitinib, erlotinib, and celecoxib were observed [39,44]. In colorectal, gastric, liver, oral, and prostate cancer cell lines, synergistic anti-cancer activities were observed with tocotrienols and drugs commonly used in breast cancer treatment—docetaxel, paclitaxel, doxorubicin, or capecitabine [39]. Of the studies that investigated the use of tocotrienols alongside breast cancer treatments, few used relevant models.

### 4.2. Multi-Agent Chemotherapy Trends

Harlan, Enewold and Stevens described changes in early-stage breast cancer treatment between 1990 and 2010 [50]. In 1990, cyclophosphamide, methotrexate and 5-fluorouracil (CMF) were the most commonly prescribed combination of chemotherapeutic agents, irrespective of node or hormone receptor status [50]. This was short-lived because by 2005, less than 1% of all patients had received CMF alone or in combination [50]. At the same time, a distinct shift to regimens containing anthracyclines was seen in 2000, peaking in 2005 [50]. Likely in part due to cardiotoxicity, physicians shifted away from anthracycline-containing regimens, and by 2010, the preferred combination was that of cyclophosphamide plus a taxane [50]. Over the past few decades, the development of taxanes has been considered to be the main improvement in chemotherapy treatments [51]. Using six Cancer Intervention and Surveillance Network (CISNET) models to assess the impact of screening and adjuvant treatment on U.S. breast cancer mortality, advancements in chemotherapy were calculated to have had a mean contribution in mortality reduction of 38% between 2000 and 2012 [51].

### 4.3. Endocrine Therapy Trends

The vast majority (60–75%) of breast cancers are classified as estrogen-receptor positive (ER+) or progesterone receptor positive (PR+) [52]. The overall use of endocrine therapy increased between 1990 and 2010 in women with node-negative, ER-positive tumors [50]. With a 45% lower risk of breast cancer, tamoxifen was a standard chemopreventative treatment for a number of years [50]. Of women with node-positive cancer receiving endocrine therapy, only 59.4% received tamoxifen in 1990 compared to 71.5% in 2000 and 31.3% in 2010 [50]. A similar trend was seen in node-negative patients receiving endocrine therapy, with 46.1% of women receiving tamoxifen in 1990 compared to 62.8% in 2000 and 23.3% in 2010 [50]. However, in 1994 the FDA issued a strong warning of the risk of uterine cancer, and in 2002 added a black box warning that curtailed tamoxifen use [50]. Over the next few years, the FDA approved three aromatase inhibitors: letrozole, exemestane, and anastrozole. With superior efficacy and a smaller overall side-effect profile, aromatase inhibitors were well positioned to replace tamoxifen [53]. The addition of aromatase inhibitors was the driving force behind the 29% mean contribution of advancements in hormone therapy to the mortality reduction between 2000 and 2012 [51].

### 4.4. Hormone Targeted Therapy Trends

Overexpression of HER2 is seen in roughly 15–20% of breast cancer cases [54]. HER2 positive breast cancers are known to be particularly aggressive, but a breakthrough came in 2006 when trastuzumab became available [55]. Weekly paclitaxel, carboplatin and trastuzumab neoadjuvant treatments were shown to be highly effective for women with stage II or III HER2-positive breast cancer [56]. In the phase 3 CLEOPATRA trial, the addition of pertuzumab to trastuzumab and docetaxel was superior to trastuzumab and docetaxel alone [55]. The ongoing TRAIN2 study is investigating the efficacy of pertuzumab with paclitaxel, carboplatin, and trastuzumab treatment compared to pertuzumab alongside an anthracycline-taxane-trastuzumab regimen [57]. In the CISNET models, the estimated contribution of trastuzumab to a reduction in overall mortality was 15%, but regarding the positive subtypes of HER2 it was 40% [51].

## 5. Safety

Apart from their natural sources, tocotrienols are found in food, cosmetics, and dietary supplements. In recent years, the medical applications of tocotrienols have grown, and, accordingly, are subject to government oversight. According to the Federal Food, Drug, and Cosmetic Act, the use of a food additive is contingent upon approval by the U.S. Food and Drug Administration (FDA) unless it has been deemed to be safe when used as intended by qualified experts [58]. This recognition is based upon “the application of generally available and accepted scientific data, information, or methods, which ordinarily are published, as well as the application of scientific principles, and may be corroborated by the application of unpublished scientific data, information, or methods” [58]. The Malaysian Palm Oil Board in America and American River Nutrition manufacture two of the predominantly used formulations of tocotrienols. Both companies independently submitted GRAS notifications for their tocotrienol products to the FDA. In 2010, palm tocotrienol-rich fractions produced by Malaysian Palm Oil Board in America attained GRAS status [59]. In 2014, the same became true for American River Nutrition’s DeltaGold^®^ [60]. Moreover, upon consultation of the U.S. FDA MedWatch database, no safety alerts have been issued related to the use of tocotrienols as dietary supplements [61].

One of the changes to supplement fact label regulations announced by the FDA in 2016 was the transition to reporting Vitamin E in mg of α-tocopherol [62]. While the FDA’s estimates of vitamin E requirements and intake are based solely on α-tocopherol, the European Food Safety Authority (EFSA) recognizes that the isomers differ in bioavailability and distribution. In the opinion of the Panel on Food Additives, Flavourings, Processing Aids and Materials in Contact with Food (AFC) of the EFSA, the upper limits for Vitamin E cannot be extended to a safety assessment of tocotrienols [63].

Within the FDA recommendations for deriving the maximum recommended starting dose (MRSD) in initial human clinical trials, the first step is to determine the no observed adverse effect level (NOAEL) [64]. This acknowledged safety standard is defined as the highest dose that fails to produce significant biological adverse effects in the treatment group, as compared to the control [64]. Nakamura et al. performed the first oral subchronic toxicity study, determining the NOAEL of tocotrienols in rats. After the 13-week study, they reported the NOAEL to be 120 and 130 mg/kg body weight/day for male rats and female rats respectively [65]. The AFC concurred with the aforementioned NOAEL [63]. There was insufficient data for the scientific panel to come to a conclusion on the proposed intake of a 1000 mg preparation of tocotrienols or use levels of tocotrienols, but they acknowledged that at 5 mg/kg bw/day, no adverse effects are seen in humans [63]. Several years later, a chronic animal toxicity study in rats was performed. In this yearlong study, the NOAEL was reported to be 303 mg/kg/day for males, and 472 mg/kg/day for females [66]. Furthermore, in a summary of sixteen major clinical studies on tocotrienols in humans, only one study reported a few subjects experienced symptoms associated with tocotrienol supplementation [67].

## 6. Bioavailability

### 6.1. Plasma

Shen et al. were the first to disclose robust evidence for the safety of multiple dosages of supplemented tocotrienols [68]. In this subchronic, double-blinded placebo-controlled randomized study, postmenopausal osteopenic women were given a placebo, 300 mg, or 600 mg of annatto tocotrienols daily [68]. Using blood test parameters, the authors concluded that a daily dose of 600 mg tocotrienol for three months was safe [68]. Serum concentrations of all eight vitamin E isomers was documented at 0, 6, and 12 weeks; significantly increased levels were only seen for δ-tocotrienol in the supplemented groups [68]. With continued supplementation, the higher levels of δ-tocotrienol remained elevated by the end of the study and reflected the composition of the supplement [68].

An individual’s food status has been shown to greatly influence the pharmacokinetic and bioavailability of tocotrienols. Following the administration of a mixed 300 mg supplement, the pharmacokinetic parameters of δ-, γ-, and α-tocotrienols in healthy adults were measured under fed and fasted conditions [69]. Without food, the maximum concentration, volume of distribution, and AUC0-∞ were 137.7 ± 60.9 ng mL^−1^, 433.0 ± 208.5 Vd/f, and 581.6 ± 288.3 h ng mL^−1^, respectively [68]. When taken with a high-fat meal, the pharmacokinetic parameters were significantly different at 341.8 ± 92.0 ng mL^−1^, 130.2 ± 58.4 Vd/f, and 1433.4 ± 321.4 h ng mL^−1^, respectively [69]. Increases in pharmacokinetic parameters were also seen in γ- and α-tocotrienols [69]. Food increased bioavailability and more than doubled tocotrienol absorption [69].

The first human study to assess the pharmacokinetic and bioavailability of pure tocotrienols assessed the effects of 125, 250 and 500 mg/d doses of annatto-based δ-tocotrienol under fed conditions [70]. Plasma concentration levels of all eight vitamin E isomers were quantified at 0, 1, 2, 3, 4, 6, 8, and 10 h post administration [70]. For all three doses, the time to reach maximum plasma concentration ranged between 3 and 4 h for tocotrienols but between 3 and 6 h for tocopherols [70]. Dose-dependent increases in pharmacokinetic parameters were seen for δ-tocotrienol as well as for other isomers [70]. The half-lives of the tocotrienol group ranged between 1.39 and 4.39 h versus 1.82 and 5.22 h for the tocopherol group [70]. The authors concluded that pure tocotrienols have superior bioavailability over tocopherols and tocol mixtures [70]. In addition, this study provided further documentation of the stepwise bioconversion of tocotrienols to tocopherols [70]. In the HPLC analysis, δ, γ, and α-tocotrienols and δ-tocopherol were not detected at 0 h [70]. The profile showed that for the 125, 250 and 500 mg/d doses of annatto-based tocotrienols, a small δ-tocotrienol peak appeared at 2 h, with a maximum at 3 h, which is also when δ-tocopherol appeared [70]. The authors proposed that δ-tocotrienol was converted to γ-, β-, and α-tocotrienols followed by reduction to δ-, γ-, β-, and α-tocopherols [70].

Daily dietary consumption of tocotrienols is estimated to be only several milligrams, which is well below the levels necessary to render health benefits [67]. While all tocols are absorbed in the lumen of the small intestine, absorption of the different isomers varies and is highly dependent on food status. Evidence continues to accumulate supporting the safety and efficacy of pure tocotrienols.

### 6.2. Organ and Tissue

Once a drug circulates throughout the body, it is distributed into organs and tissues. Drug distribution depends on numerous factors, and the dose each organ or tissue receives varies. One study measured the accumulation of annatto tocotrienols and alpha-tocopherol in 18 different organs and tissues of laying hens [71]. The four treatment groups (control, 2000 mg/kg annatto tocotrienols, 2000 mg/kg annatto tocotrienols + 200 mg/kg alpha-tocopherol, 2000 mg/kg annatto tocotrienols + 1000 mg/kg alpha-tocopherol) received supplemented feed for seven weeks [71]. Between the four treatment groups, significant differences in tocol levels were seen in the fat pad, liver and gall bladder, oviduct, forming yolks, laid yolks, kidney, brain, thigh, and breast [71]. Notably, significantly less gamma-tocotrienol was seen in the 2000 mg/kg annatto tocotrienols + 1000 mg/kg alpha-tocopherol group compared to the control or 2000 mg/kg annatto tocotrienols group [71]. The authors deduced that alpha-tocopherol differentially influences gamma-tocotrienol by hindering its transfer to laid and forming yolks [71]. Numerous other groups have documented interference in the accumulation of tocotrienols by alpha-tocopherol.

Patel et al. were the first to demonstrate that supplemented tocotrienols are transported to vital organs [72]. Healthy patients took 400 mg α-tocopherol daily for twelve weeks [72]. Surgical patients (heart or liver transplant recipients, reconstructive plastic surgery in morbidly obese patients, or recalcitrant epileptic patients undergoing resection) received 400 mg mixed tocotrienols daily, ideally for at least four full weeks prior to surgery [72]. Vitamin E levels were measured in whole blood, skin, abdominal adipose, tissue, brain, cardiac muscle, and liver [72]. Tocotrienols accumulated in vital organs, even in the shortest durations of supplementation [72]. After 6 and twelve weeks of supplementation, the mean whole blood concentration of α-tocotrienol were measured to be >1.5 and 2.5 μmol/L, respectively [72]. These levels are 20 times higher than the previously reported concentration at which potent neuroprotective benefits are seen, 100 nM [43].

Perhaps most relevant to the topic is a study measuring tocotrienol levels in adipose breast tissue. Palm oil, a natural source of tocotrienols, is the primary fat consumed by Malaysians. As it was previously estimated that Malaysians consume 10–15 mg of vitamin E daily, the patients’ diets were not supplemented further [73]. Samples were taken from 75 Malaysian patients with breast lumps—40 of the women had malignancies; the remaining patients had benign pathologies [73]. The authors noted that the distribution of the tocotrienol isomers reflected that of the dietary composition [73]. While there was extensive variability in alpha-tocopherol levels, the mean content of β-, γ-, and δ-tocopherols in malignant and benign lumps was 11.5 and 9.59 µg/g, respectively [73]. A significant difference in the mean content of α-, γ-, and δ- tocotrienols was seen, measuring 20.1 and 13.7 µg/g in malignant and benign lumps, respectively [73]. Based on previous in vitro and in vivo studies demonstrating the protective effects of tocotrienols, the authors hypothesized that its elevated levels in patients with benign lumps compared to those with malignant lumps could be due to protective effects [73].

## 7. Clinical Trials

Clinical trial results for the use of tocotrienols in breast cancer patients are extremely limited; however, a novel study in Malaysia investigated the effects as an adjuvant in the treatment of early breast cancer with tamoxifen (Table 2) [74]. The 240 study participants had been diagnosed with Stage I or II tumor-node metastasized breast cancer or estrogen-receptor positive tumors [74]. The women received daily treatments of tocotrienol-rich fraction (TRF) plus tamoxifen or a placebo plus tamoxifen treatment [74]. The α-tocopherol and α-, γ-, and δ-tocotrienols plasma levels in the group receiving TRF plus tamoxifen were significantly higher than the plasma levels for the placebo plus tamoxifen group [74]. Six patients in the placebo group were confirmed to have succumbed to breast cancer while 20 experienced a recurrence [74]. In the intervention group, two patients passed away from breast cancer and 16 experienced recurrence [74]. After adjusting for age, ethnicity, stage, and lymph node status, the risk of mortality was 60% lower for patients in the intervention group than the placebo-plus-tamoxifen group [74]. While this result was nonsignificant, the authors acknowledged the limitations of the small sample size, overly broad primary outcome, and lack of randomization [74]. In light of their results, Nesaretnam et al. concluded that further clinical studies of tocotrienols as adjuvants in breast cancer patients are warranted [74].

A phase II open-label was completed at the Fondazione IRCCS Istituto Nazionale Tumori in Milan, Italy, in 2017 (Table 2) [76]. In this study, 50 women with primary breast cancer received 200 mg b.i.d. of δ-tocotrienol for the four weeks leading up to surgery [76]. The effects––oxidant and antioxidant capacity, anti-inflammatory activity, proliferation, apoptosis, and immune response––were measured [76]. While the results have yet to be posted, this study may offer insights into the antioxidant and anti-inflammatory capacity of tocotrienols in a preoperative setting.

More recently, a study of 80 female patients was conducted at the Department of Oncology at the Vejle hospital in Denmark and concluded in 2019 (Table 2) [77]. In this phase II trial, the ability of tocotrienols to enhance the efficacy of neoadjuvant chemotherapy while reducing side-effect profiles before surgery was investigated [77]. Based on HER2 status, chemotherapeutic regimens included epirubicin, docetaxel or paclitaxel, cyclophosphamide, trastuzumab, and ertuzumab [77]. Tocotrienols supplements (300 mg) were given three times a day in combination with the neoadjuvant therapy [77]. While the results have yet to be posted, this trial will further our understanding of the potential of tocotrienols in breast cancer treatment [77].

## 8. Bibliographic Terms over Time

**Figure 3:**
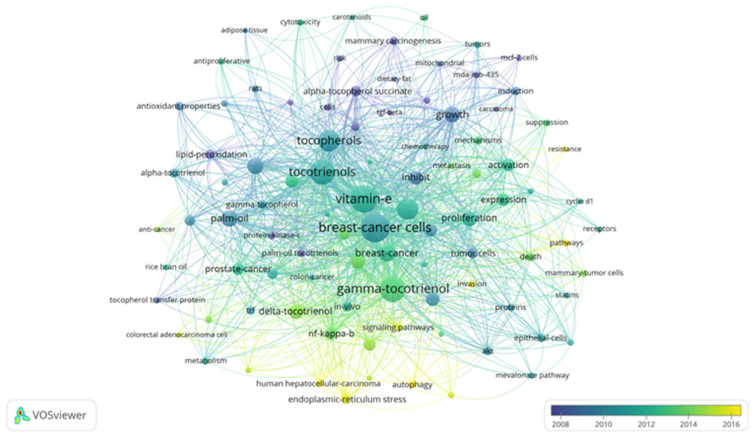
Term map for years 2008–2016 generated from Search 1. Visualization of 96 terms that occurred a minimum of 5 times in publications. Table S2 lists the visualized terms and their respective occurrences, average year of publication, and average number of citations.

**Figure 4:**
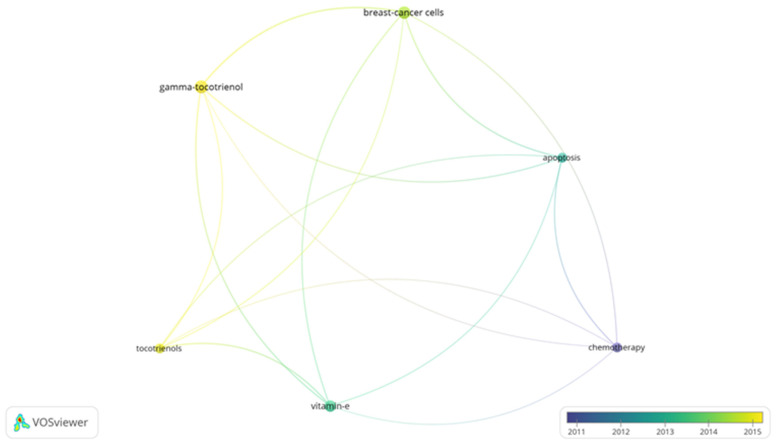
Term map for years 2011–2015 generated from Search 2. Visualization of 6 terms that occurred a minimum of 5 times in publications. Table S3 lists the visualized terms and their respective occurrences, average year of publication, and average number of citations.

**Figure 5:**
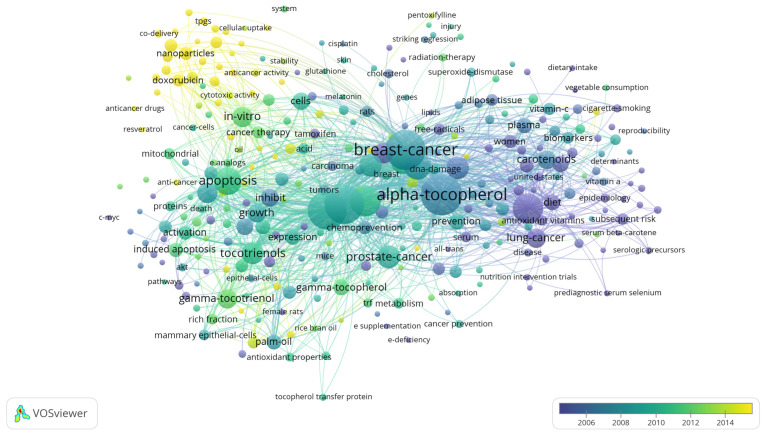
Term map for years 2005–2015 generated from Search 3. Visualization of 311 terms that occurred a minimum of 5 times in publications. Table S4 lists the visualized terms and their respective occurrences, average year of publication, and average number of citations.

**Figure 6:**
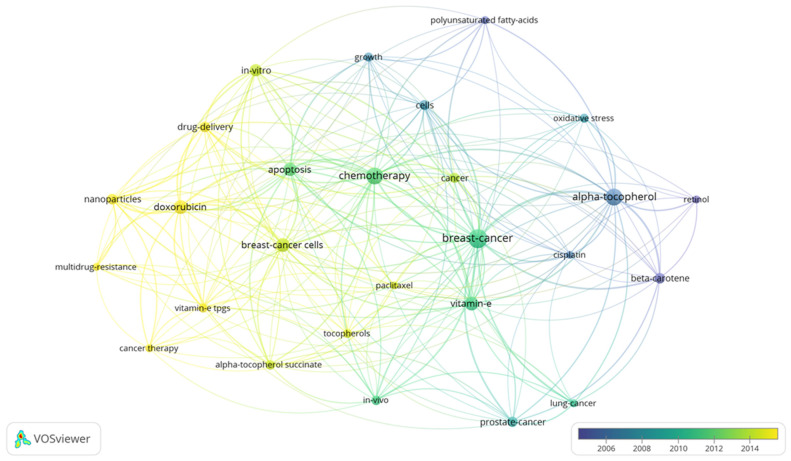
Term map for years 2005–2015 generated from Search 4. Visualization of 27 terms that occurred a minimum of 5 times in publications. Table S5 lists the visualized terms and their respective occurrences, average year of publication, and average number of citations.

Bibliometrics are increasingly popular methods for analyzing scientific publications on a macroscale [24]. Applicable to scientists, institutions, journals, and countries, it gives insight into research productivity, discipline evolution, research relevancy, and contributions to scientific fields [24,78]. As the number of scientific publications increases, so does the utility of bibliometric methods [24]. To guide future studies, this review used bibliometric analysis to highlight trends in research surrounding the use of tocotrienols in breast cancer treatments. From the analysis, the networks generated with VOSviewer are shown in Figure 3, Figure 4, Figure 5 and Figure 6.

A search limited to tocotrienols and breast cancer returned 96 terms in 5 clusters, 1983 links, and a total link strength of 6486 (Figure 3). The 5 clusters were inferred to be early research, molecular mechanisms, in vivo studies, vitamin E in related cancer, and centered on the cell cycle. The top 5 terms were “breast-cancer cells”, “vitamin-e”, “gamma-tocotrienol”, “tocotrienols, and “apoptosis.” A focused search on tocotrienols, breast cancer, and chemotherapy produced 6 terms in 2 clusters, 15 links, and a total link strength of 40 (Figure 4). The top 5 terms were nearly identical to those of the previous search: “breast-cancer cells”, “gamma-tocotrienol”, “apoptosis”, “vitamin-e”, and “tocotrienols.” In comparison, a search for tocopherols and breast cancer returned 311 terms in 6 clusters, 9032 links, and a total link strength of 21,431 (Figure 5). The 6 clusters were inferred to be risk factors and prevention, pharmacodynamics and pharmacokinetics in anticancer research, in vitro and in vivo studies, prevention and supplementation, clinical trials, and preclinical trials. To emphasize the point further, a search for tocopherols, breast cancer, and chemotherapy yielded 27 terms in 3 clusters, 216 links, and a total link strength of 488 (Figure 6). The last 3 clusters were inferred to be chemotherapeutic treatment, vitamin E in cancer, and in vivo studies.

## 9. Concluding Remarks

As the most commonly diagnosed cancer and the leading cause of cancer death for women, breast cancer, carries a tremendous global burden [1]. From 1975 to 2010, over 70% of women with node-positive Stage IIb, ER+/HER2-negative breast cancer (ages 50–69) received both multi-agent and hormonal treatment [51,79]. Even with recent advancements, a reduced quality of life, limited efficacy, and significant side effects still accompany standard treatments. Tocotrienols have shown tremendous promise as an anticancer adjuvant agent, even acting synergistically with chemotherapies. However, there is a lack of knowledge regarding their use alongside breast cancer treatments––more specifically, a lack of clinically relevant models. The discrepancy between tocopherol and tocotrienols research in breast cancer was further emphasized in a bibliometric analysis (Figure 3, Figure 4, Figure 5 and Figure 6). In light of the aforementioned trends in breast cancer treatment, models using taxanes, aromatase inhibitors, and hormone-targeted treatments are needed.

## Figures and Tables

**Table 1:** Web of science search results prior to bibliometric analysis.

Search	Years	Total Publications	Total Citing Articles
Search 1	1945–2021	246	5821
Search 2	1945–2021	13	282
Search 3	1945–2021	750	24,670
Search 4	1945–2021	66	1562

**Table 2:** All interventional clinical trials of tocotrienols in breast cancer patients [74,75,76,77]. Accessed 28 May 2021.

ClinicalTrials.gov Identifier	Intervention	Pathology	Treatment	Phase	No. of Patients	Dose; Duration	Outcome/Status
NCT01157026	TRF	ER+ or PR+	Tamoxifen	Pilot	240	200 mg; daily/five years	Completed, 2010
NCT04496492	δ-Tocotrienol	Primary Breast Cancer	Preoperative observation	Phase II	50	200 mg; 2×/day for 4 weeks	Completed, 2017
NCT02909751	Tocotrienol	Breast adenocarcinoma	Neoadjuvant chemotherapy	Phase II	80	300 mg × 3 daily	Completed, 2019
NCT03855423	Tocovid Suprabio	Operable breast cancer	Preoperative observation	Phase Ib	12	3 + 3 step up design	Unknown

## Data Availability

The data presented in this study are available in Table S1: Thesaurus File. Table S2: Term Map Data, Search 1. Table S3: Term Map Data, Search 2. Table S4: Term Map Data, Search 3. Table S5: Term Map Data, Search 4.

## Supplementary Materials

The following are available online at https://www.mdpi.com/article/10.3390/antiox10091383/s1, Table S1: Thesaurus File. Table S2: Term Map Data, Search 1. Table S3: Term Map Data, Search 2. Table S4: Term Map Data, Search 3. Table S5: Term Map Data, Search 4.
